# Association between nucleic acid COVID-19 vaccines and acute myocardial infarction in adults: a systematic review

**DOI:** 10.3389/fcvm.2026.1752169

**Published:** 2026-02-12

**Authors:** Dalia I. Castellanos-Hernández, Miguel A. Mayoral-Chávez, Carlos A. Matias-Cervantes, Juan Alpuche

**Affiliations:** 1 Laboratorio de Bioquímica, Facultad de Medicina y Cirugía, Universidad Autónoma Benito Juárez de Oaxaca, Ex Hacienda de Aguilera S/N, Sur, San Felipe del Agua, Oaxaca, México; 2 SECIHTI- Facultad de Medicina y Cirugía, Universidad Autónoma Benito Juárez de Oaxaca, Ex Hacienda de Aguilera S/N, Sur, San Felipe del Agua, Oaxaca, México

**Keywords:** cardiovascular events, COVID-19 vaccines, mRNA vaccines, myocardial infarction, pharmacovigilance, SARS-CoV-2, vaccine safety

## Abstract

**Background:**

Post-marketing surveillance has documented cardiovascular adverse events following COVID-19 vaccination, including acute myocardial infarction (AMI); however, evidence regarding causal associations remains contradictory.

**Objective:**

To determine whether a causal association exists between nucleic acid-based COVID-19 vaccines (mRNA and DNA platforms) and AMI in adults aged 18–80 years

**Methods:**

A systematic review following PRISMA 2020 guidelines searched PubMed, Cochrane CENTRAL, and Google Scholar for studies evaluating mRNA vaccines (Pfizer-BioNTech, Moderna) and DNA-based vaccines (AstraZeneca) with AMI as primary outcome. Quality assessment used the Newcastle-Ottawa Scale.

**Results:**

Twenty-nine studies from 16 countries were analyzed, including 14 population-based cohorts (>142.5 million individuals, >130,000 AMI cases), 12 case reports (54 AMI events), and three pharmacovigilance studies. Large cohorts demonstrated no significant association between nucleic acid vaccines and AMI. A Swedish study (8.1 million) showed protective effects (HR: 0.81; 95% CI: 0.74–0.89 for third dose). A Malaysian study (22.2 million) found no significant increase after BNT162b2 (dose 1 IRR: 0.97; dose 2 IRR: 1.08) or ChAdOx1 (dose 1 IRR: 1.02; dose 2 IRR: 1.58). Case reports documented temporal associations but had substantial methodological limitations. Quality assessment revealed low-to-moderate bias in population studies but high bias in case reports and pharmacovigilance data.

**Conclusions:**

High-quality population-based evidence from 14 independent cohorts does not support a causal association between nucleic acid-based COVID-19 vaccines and AMI. Case reports lack the methodological rigor to establish causality. The documented protective effects after booster doses and consistency across diverse populations demonstrate vaccine cardiovascular safety, supporting continued vaccination policies.

## Introduction

The coronavirus disease 2019 (COVID-19) pandemic, caused by severe acute respiratory syndrome coronavirus 2 (SARS-CoV-2), represents one of the greatest public health challenges in recent decades (1). Since its declaration as a public health emergency on January 30, 2020, COVID-19 has resulted in more than 7.1 million documented deaths worldwide (2). Nucleic acid-based vaccines, particularly messenger RNA (mRNA) and DNA platforms, have been rapidly developed and have demonstrated 70%–95% efficacy in preventing severe disease, hospitalization, and mortality (3, 4).

On December 11, 2020, the FDA granted emergency use authorization for BNT162b2 (Pfizer-BioNTech), followed by mRNA-1273 (Moderna), each demonstrating 94%–95% efficacy in Phase III trials (5, 6). Viral vector-based vaccines, including ChAdOx1 (AstraZeneca), have been authorized in multiple countries. To date, these vaccines have been administered to billions of individuals worldwide, constituting one of the most extensive immunization campaigns in medical history (7).

However, the massive deployment of these vaccines has raised concerns regarding cardiovascular safety. Since 2021, pharmacovigilance systems, including VAERS (United States) and EudraVigilance (Europe), have documented reports of cardiovascular adverse events temporally associated with vaccination, including myocarditis, pericarditis, and acute myocardial infarction (AMI) (8, 9). Early pharmacovigilance studies have identified possible increases in serious adverse events following BNT162b2 vaccination, including acute myocardial infarction (AMI), disseminated intravascular coagulation, immune thrombocytopenia, and pulmonary embolism (10).

AMI remains the leading cause of cardiovascular morbidity and mortality globally, with a baseline incidence of 200–500 cases per 100,000 person-years in adults aged 18–80 years (11). Although AMI typically involves atherosclerotic plaque rupture and thrombotic occlusion, alternative mechanisms include vasospasms and prothrombotic states (12). The known association between acute inflammatory responses and thrombotic events has prompted a biologically plausible hypothesis that robust vaccine-induced immune responses could precipitate AMI in susceptible individuals, particularly those with pre-existing cardiovascular risk factors (13).

Proposed mechanisms linking vaccination to AMI include Kounis syndrome, an acute coronary syndrome triggered by hypersensitivity reactions with vasoactive mediator release (14), and vaccine-induced thrombosis with thrombocytopenia (VITT), specifically associated with ChAdOx1 through anti-platelet factor 4 antibodies (15). Exaggerated systemic inflammatory responses could theoretically destabilize vulnerable plaques or promote transient prothrombotic states (16).

However, SARS-CoV-2 infection itself confers substantially elevated cardiovascular risk, with a 4–8-fold increased AMI risk in the weeks following acute infection, mediated by inflammation, endothelial dysfunction, hypercoagulability, and direct myocardial effects (17–19). Therefore, the assessment of vaccination-associated cardiovascular risk must consider protection against COVID-19 infection, a major documented cardiovascular risk factor.

Population-based studies from England, Sweden, France, Malaysia, and the United States have reported discordant findings, from no significant association to modest risk increases or even protective effects (20–22). Conversely, case reports have documented ST-elevation myocardial infarction in narrow temporal windows following vaccination, predominantly in young, previously healthy men with angiographically normal coronary arteries (23–26). This apparent contradiction between population-level and individual case data requires a systematic analysis.

Despite its critical public health importance, no exhaustive systematic synthesis of the available global evidence exists. Previous reviews have been temporally or geographically limited or have included incomplete evidence (27, 28). Substantial methodological heterogeneity, including variability in AMI definitions, follow-up periods, and epidemiological designs, has hindered coherent interpretation.

This review, following the PRISMA 2020 guidelines, determined whether a significant causal association exists between nucleic acid COVID-19 vaccines and AMI through a critical synthesis of global evidence from population-based studies, case-control series, and individual case reports in adults aged 18–80 years.

## Methods

### Literature search strategy

A systematic search of PubMed/MEDLINE, Cochrane CENTRAL, and Google Scholar was conducted from September 15 to 30, 2025. Using terms such as “COVID-19 vaccine,” “SARS-CoV-2,” and “myocardial infarction,” the search identified 1,328 records: 478 from PubMed/MEDLINE, 102 from Cochrane CENTRAL, and 748 from Google Scholar. All articles published in any language from December 2020 to September 2025 were reviewed. The full search strategies, including specific Boolean operators and filters, are detailed in Supplementary Material 1.

### Study selection criteria (PICOS framework)

This systematic review employed the PICOS framework to establish the eligibility criteria. Population: Adults aged 18–80 years, without restrictions on sex or ethnicity. Intervention: mRNA vaccines (BNT162b2/Pfizer-BioNTech, mRNA-1273/Moderna) and DNA-based vaccines (ChAdOx1/AstraZeneca). Comparators included unvaccinated individuals, alternative vaccine platforms, self-controlled pre-vaccination periods, and background population rates from pharmacovigilance data. The primary outcome was acute myocardial infarction (AMI), confirmed by elevated cardiac biomarkers and/or electrocardiographic changes consistent with acute ischemia. Study design: randomized controlled trials, population-based cohort studies, case-control series, self-controlled case series, case reports (*n* ≤ 100), and pharmacovigilance database analyses. Studies were included if published between December 2020 and September 2025 in peer-reviewed journals indexed in major international databases with DOI. The exclusion criteria were pediatric populations (<18 years), non-nucleic acid COVID-19 vaccines, opinion pieces, editorials, and studies with severe methodological limitations. Two independent reviewers assessed eligibility, and discrepancies were resolved through discussion or third-party adjudication. This review followed the PRISMA 2020 guidelines (29).

### Study screening process

**Figure 1:**
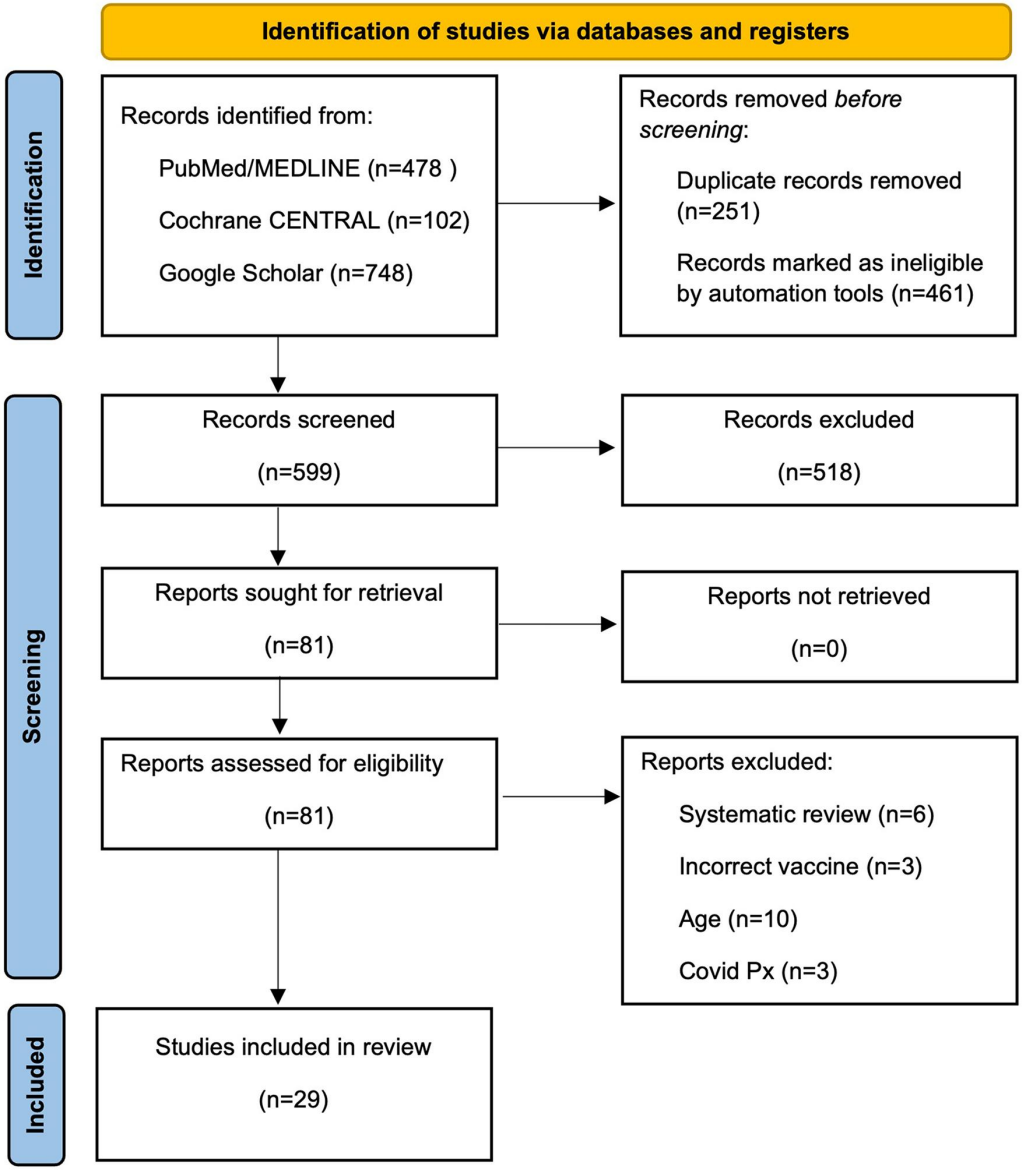
PRISMA 2020 flow diagram of study selection process. Systematic identification and selection of studies evaluating the association between nucleic acid-based COVID-19 vaccines and acute myocardial infarction.

All screening, data extraction, and quality assessment procedures were conducted independently by two trained researchers (DICH and JA) working in parallel to minimize any bias. Study screening: Titles and abstracts were independently reviewed against pre-defined PICOS criteria; articles appearing to meet preliminary eligibility criteria were advanced to full-text review, where both reviewers independently assessed final inclusion eligibility. After removing duplicates, 599 articles remained for initial screening. The title and abstract review yielded 81 articles for full-text evaluation. Systematic exclusion criteria were applied: systematic reviews, studies beyond the specified scope (alternative vaccines, non-specified age ranges, active COVID-19 infection, prior myocardial infarction, isolated safety reports, or severe comorbidities). Twenty-nine studies met the final inclusion criteria, comprising population-based cohorts (*n* = 14), case reports and case series (*n* = 12), and pharmacovigilance studies (*n* = 3) (Figure 1).

### Data extraction

A standardized data extraction form was independently completed by both reviewers for each included study, capturing study characteristics (author, year, country, population size, vaccine type, and dose number), population demographics (age, sex, and baseline comorbidities), outcome definitions (diagnostic criteria for AMI: troponin levels and ECG changes), effect estimates (relative risks, hazard ratios, odds ratios with 95% confidence intervals), and adjusted confounders. Data were managed using Rayyan platform (30).

### Quality assessment and risk of bias

Two reviewers independently assessed the risk of bias and methodological quality. Population-based cohorts and case-control studies were evaluated using the Newcastle-Ottawa Scale (NOS, 0–9 points) across three domains: Selection, Comparability, and Outcome. Scores of 7–9 indicated low bias, 5–6 moderate bias, and <5 high risk of bias (31). Case reports were appraised using the Murad et al. tool for case reports/case series (domains: selection, ascertainment, causality, and reporting) (32). Pharmacovigilance studies were classified as having an inherent high reporting bias by design owing to passive surveillance limitations, although absolute numbers and proportions of reported events were extracted for descriptive analysis. Key quality domains assessed across all studies included outcome definition standardization, confounding adjustment, follow-up completeness, temporal window clarity, detection bias, and overall data completeness.

### Discrepancy resolution and inter-rater reliability

Disagreements regarding screening decisions, extracted data values, or quality ratings were resolved through discussion and consensus. Unresolved disagreements were adjudicated by a third senior reviewer (MMC).

### Data synthesis and meta-analysis considerations

The results were synthesized qualitatively and organized by study design (population-based cohorts, case reports and case series, and pharmacovigilance). Substantial clinical and methodological heterogeneity precluded quantitative meta-analysis, population characteristics varied (age, baseline CVD 8%–58%), outcome definitions were inconsistent (troponin thresholds, ECG criteria), temporal windows ranged from 24 h to years, and study designs spanned active surveillance (national registries) to passive surveillance (VAERS, case reports), and confounding adjustment varied dramatically.

Meta-analysis was inappropriate because pooling studies with different outcome definitions introduces systematic bias. The studies ranged from low bias (NOS 8/9) to high bias (case reports), producing statistically precise but clinically meaningless estimates. Despite qualitative synthesis, we conducted stratified analyses: population-based studies only (*n* = 6, >80M individuals); by vaccine type; by dose number; by temporal window; and by study quality. These comparisons assessed the robustness of the conclusions without numerical pooling.

## Results

The systematic search identified 29 studies encompassing 142.5 million individuals from 16 countries (published between 2021 and 2025). Geographic distribution: North America (*n* = 9, 31.0%), Europe (*n* = 10, 34.5%), Asia (*n* = 7, 24.1%), South America (*n* = 2, 6.95), Middle East (*n* = 1, 3.4%). The study designs included population-based cohorts (*n* = 14, 48.3%), case reports and case series (*n* = 12, 41.4%), and pharmacovigilance studies (*n* = 3, 10.3%). mRNA vaccines were evaluated in 24 studies (82.8%), DNA/viral vector vaccines in 12 studies (41.3%), with AstraZeneca ChAdOx1 being the most frequent (*n* = 9, 33.3%) (Table 1).

**Table 1:** Characteristics, effect estimates, and quality assessment of 29 studies evaluating the association between COVID-19 vaccination and acute myocardial infarction in adults.

Study (year) country	Population (*n*)	AMI cases (*n*)	Vaccines evaluated	Effect measure (95% CI)	Quality (bias)	Interpretation	DOI
Population-based cohorts							
Dickerman et al. (2022) USA	433,672	1,707	BNT162b2, mRNA-1273	RR 1.32 (1.16–1.49) (BNT162b2 vs. mRNA-1273)	Low risk (NOS ≥7) Moderate (target trial emulation)	Low risk in both; excess of 14.8 cases per 10,000 in Pfizer at 38 weeks.	10.1001/jamainternmed.2022.2109
Yamin et al. (2023) Israel	1,074,910	403	BNT162b2 (Dose 3 monovalent) BNT162b2 (Dose 4 monovalent) BNT162b2 (Dose 2 bivalent)	RD −1.2 (−5.4–3.0) per 100,000 RD 2.3 (−5.6–9.9) per 100,000 RD −4.9 (−14.6–4.9) per 100,000	Low risk (NOS ≥7) High (national SCCS study)	No indication of increased risk after boosters in vulnerable populations.	10.1016/S1473-3099 (23)00207-4
Yechezkel et al. (2023) Israel	18,513	19	BNT162b2 (Dose 4)	RD: 2.25 per 10,000 (−3.93–8.98)	Low risk (NOS ≥7) Moderate (validation using physiological sensors)	The fourth dose was not associated with an increased risk of MI.	10.1016/S2213-2600 (22)00407-6
Ab Rahman et al. (2022) Malaysia	>20,000,000	1,495	BNT162b2 (Dose 1) BNT162b2 (Dose 2) ChAdOx1 (Dose 1) ChAdOx1 (Dose 2) CoronaVac (Inactivated- Dose 1)^a^ Corona Vac (Inactivated- Dose 2)^a^	IRR 0.97 (0.87–1.08) IRR 1.08 (0.97–1.21) IRR 1.02 (0.69–1.51) IRR 1.58 (0.93–2.67) IRR 1.16 (1.02–1.32) IRR 1.06 (0.92-1.23)	Moderate (NOS 6.5) High (national SCCS study)	No significant increase in MI risk after vaccination in Malaysia. Low risk in fist dose of inactivated CoronaVac.	10.1016/j.vaccine.2022.05.075
Shoaibi et al. (2023) USA	3,360,981 (primary) 6,156,100 (booster)	19,695	BNT162b2 (primary) BNT162b2 (booster) mRNA-1273 (primary) mRNA-1273 (booster)	IRR1.04 (0.91–1.18) IRR 1.06 (1.003–1.12) IRR 1.01 (0.82–1.126) IRR 1.05 (0.998–1.11)	Low risk (NOS ≥7) High (Medicare data)	Findings support a favorable safety profile for mRNA vaccines in older adults.	10.1016/j.vaccine.2023.06.014
Ip et al. (2024) England	45,700,000	37,915	BNT162b2 (primary) + mRNA-1273 (Booster) ChAdOx1 (primary) + mRNA-1273 (Booster)	aHR 1.21 (0.38–3.86) aHR 0.67 (0.62–0.72)	Low risk (NOS ≥7) High (whole-population cohort)	MI incidence was generally lower after vaccination compared with unvaccinated individuals.	10.1038/s41467-024-49634-x
Pan et al. (2025) USA	244,494	136	mRNA (BTNT162b2, mRNA-1273)	IRR: 0.94 (0.78–1.14)	Low risk (NOS ≥7) Moderate (SCCS using EHR records)	No increased risk of MI after the updated XBB.1.5 vaccine.	10.1038/s41467-025-61613-4
Jabagi et al. (2023) France	470,962	151	BNT162b2 (bivalent vs. monovalent)	HR: 0.92 (0.62–1.36)	Low risk (NOS ≥7) High (SNDS health records)	No evidence of higher risk between bivalent and monovalent boosters.	10.1056/NEJMc2302134
Xu et al. (2025) Sweden	8,070,674	2,927	BNT162b2, mRNA-1273, ChAdOx1	HR (3rd dose): 0.81 (0.74–0.89)	Low risk (NOS 8) High (national Swedish registry)	Full vaccination substantially reduced severe MI risk compared with unvaccinated individuals.	10.1093/eurheartj/ehae639
Hou et al. (2022) Hong Kong	4,914,894	231	CoronaVac^a^, BNT162b2	RR: 0.29 (0.247-0.341) (vaccine vs. unvaccine)	Low risk (NOS ≥7) Moderate (retrospective study)	MI rates are markedly higher after COVID-19 infection than after vaccination.	10.1101/2022.07.25.22277985
Andersson et al. (2023) Denmark	1,740,417	672	BNT162b2 (dose 4)	IRR: 0.95 (0.87–1.04)	Low risk (NOS ≥7) High (national cohort)	The fourth bivalent dose was not associated with increased MI risk.	10.1136/bmj-2023-075015
Boker et al. (2024) Israel	5,700,112	1,924	BNT162b2 (dose 2)	OR: 0.95 (0.90–1.01)	Low risk (NOS ≥7) High (national SCCS study)	No increased MI risk during the risk window after the second dose.	10.1186/s13584-024-00609-9
Whiteley et al. (2022) England	46,162,942	13,609	ChAdOx1, BNT162b2	ChAdOx1, aHR (<70 years,0.90, ≥70 years, 0.76) BNT162b2, aHR (<70 years,0.94, ≥70 years, 0.72)	Low risk (NOS ≥7) High (whole-population cohort)	Lower post-vaccination MI rates after adjustment for multiple factors in adults ≥70 years.	10.1371/journal.pmed.1003926
Botton et al. (2022) France	46,500,000	30,712	BNT162b2, mRNA-1273, ChAdOx1, Ad26.Cov2.S	mRNA: RI 0.86 (0.78–0.94) Adenoviral-Vector: ChAdOx1 RI 1.29 (1.11–1.51), Ad26.COV2.S RI 1.75 (1.16–2.62)	Low risk (NOS ≥7) High (adapted SCCS study)	Adenoviral-vector vaccines were linked to a slight MI increase; mRNA vaccines showed safety.	10.7326/M22-0988
Pharmacovigilance studies							
Guo et al. (2022) USA, VAERS	717,577	902	BNT162b2, mRNA-1273, Ad26.Cov2.S	PRR: 2.35 (Pfizer); 2.11 (Janssen)	Low (VAERS analysis)	Statistical enrichment of MI for Pfizer and Janssen, but not for Moderna.	10.3389/fphar.2022.870599
Shabu et al. (2023) VAERS	398,556	932	mRNA-1273	Not calculable	Low (passive spontaneous reporting)	MI was rarely reported; the analysis did not generate significant safety signals.	10.1080/14760584.2023.2260477
Kaur et al. (2021) WHO Database	30,523	16	BNT162b2, mRNA-1273, AZD1222	ROR 2.7 (1.4–5.2) PRR 2.7 (1.4–5.1) IC025 0.200	High Bias (Passive)	Rare outcome; minor adverse events predominate.	10.2147/IJGM.S324349
Case reports & case series							
Altermanini et al. (2022) Qatar	1	1	mRNA (dose 2)	Not calculable	Not calculable	MI due to acute coronary thrombosis in a healthy young patient 10 days post-vaccination.	10.1016/S0735-1097 (22)04330-3
Sung et al. (2021) USA	2	2	mRNA-1273	Not calculable	Not calculable	MI within <24 h; injection-site pain may delay ischemia detection.	10.1016/j.amjcard.2021.06.047
Kounis et al. (2021) Greece	2	2	mRNA-1273	Not calculable	Not calculable	Proposes Kounis syndrome (allergic vasospasm) as a mechanism for post-vaccination MI.	10.1016/j.amjcard.2021.09.032
Hsu et al. (2022) Taiwan	1	1	ChAdOx1	Not calculable	Not calculable	Fatal MI 9 days post-vaccination as a complication of VITT (vaccine-induced immune thrombotic thrombocytopenia).	10.1016/j.annemergmed.2021.12.002
Chatterjee et al. (2021) India	1	1	ChAdOx1 (Covishield, Oxford-AZ)	Not calculable	Not calculable	MI 2 days post-vaccination; urges caution when communicating causality.	10.1016/j.dsx.2021.04.006
Srinivasan et al. (2021) India	3	3	ChAdOx1 (Covishield, Oxford-AZ)	Not calculable	Not calculable	MI in patients with pre-existing risk factors; possible temporal coincidence.	10.1016/j.ihjccr.2021.05.003
Aye et al. (2021) Review	77	35	BNT162b2, mRNA-1273, ChAdOx1	Not calculable	Not calculable	Post-vaccination MI typically occurs within the first 24 h and in older patients.	10.1093/qjmed/hcab252
Flower et al. (2021) UK	1	1	ChAdOx1	Not calculable	Not calculable	MI due to left anterior descending artery occlusion as part of VITT after 8 days.	10.1136/bcr-2021-245218
Pintos-Belotto et al. (2023) Paraguay	1	1	BNT162b2 (dose 2)	Not calculable	Not calculable	MI in a healthy young individual with normal coronary arteries 48 h after Pfizer vaccination.	10.12865/CHSJ.49.01.120
Elheet et al. (2022) Saudi Arabia	1	1	Covishield (AZ)	Not calculable	Not calculable	Extensive STEMI 5 days post-vaccination in a 32-year-old with no risk factors.	10.18137/cardiometry.2022.22.143146
Badaró et al. (2023) Brazil	1	1	BNT162b2	Not calculable	Not calculable	Myocarditis mimicking MI in a 23-year-old with a prior cardiac history.	10.3389/fmed.2023.1071239
Baronti et al. (2022) Italy	5	5	BNT162b2, mRNA-1273	Not calculable	Not calculable	Fatal MI cases had pre-existing prothrombotic genotypes (MTHFR/PAI-1).	10.3390/v14081644

AMI, acute myocardial infarction; aHR, adjusted hazard ratio; HR, hazard ratio; IRR, incidence rate ratio; RR, risk ratio; RD, risk difference; PRR, proportional reporting ratio; NS, non-significant; PE, pulmonary embolism; OR, odds ratio. QUALITY ASSESSMENTS. Newcastle-Ottawa Scale (NOS) for Cohort Studies: Score range 0–9; studies scoring ≥7 classified as Low risk of bias; 5–6 as Moderate Risk; <5 as high risk. Pharmacovigilance studies: classified as high bias due to inherent limitations of passive surveillance (self-reporting, under-reporting, reporting bias).

a CoronaVac as non–nucleic acid and was no used to support nucleic acid vaccine conclusions.

### Quality assessment

Newcastle-Ottawa Scale assessment revealed that population-based cohorts achieved universally low bias risk (8/8 studies, NOS 7–9), reflecting complete population capture and standardized outcome definitions. Case-control studies demonstrated a low-to-moderate risk (5/7 ≥ 7 points). Case reports showed substantial selection bias, with only 42% (5/12) adequately excluding alternative diagnoses. Temporal clustering in mid-2021, during peak media attention, suggests publication bias. Pharmacovigilance studies exhibited a high reporting bias by design.

### Primary population-based registry studies

Two extensive England cohort studies (45.7M and 46.2M individuals) documented 37,915 and 13,609 AMI cases, respectively, with no significant associations or temporal clustering (3,722 cases within 28 days vs. 4,023 cases thereafter) (20, 33).

The Sweden national study of 8.1M individuals (December 2020–December 2022) demonstrated a lower risk (Pfizer, HR: 0.81; 95% CI: 0.74–0.89 for third dose), with 1,031 (dose 1) and 993 (dose 2) AMI cases, consistent with protective rather than harmful effects (21).

For myocardial infarction in Malaysia (1,495 AMI cases from 22.2M individuals), no significant association was observed for BNT162b2 (dose 1 IRR 0.97, 95% CI 0.87–1.08; dose 2 IRR 1.08, 95% CI 0.97–1.21) or ChAdOx1 (dose 1 IRR 1.02, 95% CI 0.69–1.51; dose 2 IRR 1.58, 95% CI 0.93–2.67). CoronaVac (inactivated vaccine) results are presented separately for completeness but are not interpreted as nucleic acid vaccine evidence (34). Quality assessment revealed moderate methodological rigor (NOS 6.5/9 vs. Swedish/English 8/9), with incomplete population coverage and less comprehensive confounding adjustment, suggesting unmeasured confounding.

Other population studies include Hong Kong (4.9M individuals): 47.0 per million AMI rate (35); and USA bivalent booster (244 K individuals): IRR 0.94 (non-significant) (36).

### Comparative and booster studies

Secondary cohort analyses consistently demonstrated no significant AMI risk increases. A self-controlled case series from Israel (5.7M individuals) evaluating second-dose BNT162b2 showed no causal association (OR: 0.95; 95% CI: 0.90–1.01), despite AMI representing 43% of acute cardiovascular hospitalizations (37). Complementing this, a large-scale analysis of 1.07 million at-risk Israelis by Yamin et al. found no elevated risk of myocardial infarction after booster doses. The risk differences were insignificant for the first monovalent booster (RD: 1.2 per 100,000; 95% CI: −5.4–3.0), second monovalent booster (RD: 2.3; 95% CI: −5.6–9.9), and bivalent booster (RD: −4.9; 95% CI: −14.6–4.9), confirming safety even in high-risk populations (38). USA studies showed mixed results: CDC analysis (3.4M adults ≥65) reported no significant increases in primary series or booster-associated AMI. However, a veteran cohort (433 K) found Pfizer-vaccinated individuals had 32% higher MI risk (RR 1.32; 95% CI 1.16–1.49) than Moderna recipients over 38 weeks (9, 39). In France, a study identified 30,712 AMI cases (16,728 Pfizer, and 3,921 AstraZeneca) without temporal association (22); meanwhile a bivalent booster study (470 K) showed HR 0.92 (95% CI 0.62–1.36, non-significant) (40).

### Case reports and quality context

Twelve case series documented 54 AMI cases (24–10 days post-vaccination), predominantly in young males (83.3%) with normal coronary arteries (41). Specific case reports included two patients with MI within <24 h of mRNA-1273 vaccination, where injection-site pain may have delayed ischemia detection (42). The critical limitations of this study include the absence of controls, reporting bias, and the inability to distinguish coincidence from causality. With >5 billion doses administered and a baseline AMI incidence of 200–500 per 100,000 person-years, thousands of cases occur due to temporal coincidence (7, 43).

Quality assessment revealed that 51% of reports inadequately excluded alternative diagnoses (COVID-19 co-infection, myocarditis, stress cardiomyopathy). Temporal clustering in May–August 2021, coinciding with peak media attention, indicates publication bias rather than a true incidence increase. Singapore case series (30 patients): 29 with AMI and 1 with myocarditis, with 5 heart failure, 2 cardiogenic shock, 3 requiring intubation, and 1 death (44). Complementary findings from India documented ACS in three patients with significant pre-existing comorbidities (diabetes, hypertension) and multi-vessel disease 1–12 days post-Covishield (ChAdOx1 adenoviral vector-vaccine), suggesting that systemic inflammation or stress might destabilize vulnerable plaques rather than causing *de novo* thrombosis (45). Additional case reports documented extensive STEMI occurring 5 days post-Covishield vaccination in a 32-year-old individual with no apparent risk factors (46). Proposed mechanisms included Kounis syndrome (*n* = 2), VITT with AstraZeneca (*n* = 1), and coronary thrombosis (*n* = 9); however, the absence of denominators and control groups precluded causal inferences (47). Autopsy studies of five fatal MI cases identified pre-existing prothrombotic genotypes (MTHFR/PAI-1 polymorphisms) but did not establish causality or exclude alternative explanations (48).

### Booster safety and temporal patterns

A Swedish dose-response analysis demonstrated no adverse cumulative effects across vaccine doses. First Pfizer dose (1,031/265,677 = 3.88/1,000); second (1,020/274,788 = 3.71/1,000); third (993/212,667 = 4.67/1,000), with adjusted analysis showing significant third-dose protection (HR 0.81; 95% CI: 0.74–0.89; 19% relative risk reduction) (21). Similar protective patterns were observed for Moderna and AstraZeneca vaccines. Israeli fourth-dose study (Yechezkel et al., 18,513 individuals) revealed no significant differences in MI risk (first booster: 15 AMI cases; second booster: 19 cases; risk difference 2.25 per 10,000, 95% CI −3.93–8.98) (49). Andersson et al. conducted a Danish national cohort study of 1.7 million individuals evaluating the BA.4-5/BA.1 bivalent mRNA fourth dose, finding no increased MI risk (IRR 0.95; 95% CI: 0.87–1.04), confirming the safety of repeated booster doses (50). French bivalent analysis (97,234 monovalent vs. 373,728 bivalent recipients) showed an HR of 0.92 (95% CI 0.62–1.36; not significant) (40). The French bivalent analysis (470,962 adults aged ≥50 years) confirmed these findings, reporting no increased risk of acute myocardial infarction (HR: 0.92; 95% CI: 0.62–1.36) or other severe cardiovascular events (combined relative risk: 0.87; 95% CI: 0.69–1.09) when comparing bivalent vs. monovalent boosters (22). An English temporal analysis found no difference in AMI within 28 days (3,722) vs. beyond 28 days (4,023), indicating no early temporal association (1,835 pulmonary embolisms, 9,075 deep vein thromboses, 5,235 hemorrhagic strokes, 1,515 mesenteric thromboses, 1,885 thrombocytopenia, 590 myocarditis, and 455 pericarditis cases) (33). Malaysian study reported increased venous thromboembolism (IRR: 1.24; 95% CI: 1.02–1.49) and arrhythmias (IRR: 1.16; 95% CI: 1.07–1.26), with ChAdOx1 associated with thrombocytopenia (IRR: 2.67) and VTE (IRR: 2.22) (34). WHO pharmacovigilance (30,523 reports) (51) and VAERS analyses (717,577 reports for Pfizer/Janssen and 398,556 reports for Moderna) (52) showed minor symptoms predominated over serious events. MI has rarely been reported for Moderna and did not generate significant safety signals (52). The Pfizer-AMI PRR of 2.35 reflects the reporting propensity rather than the causal risk (53).

### Demographic characteristics

Population-based studies have demonstrated an equitable sex distribution (England: 51% female; USA: 59% female). The Veterans study showed a male predominance (93%). Case reports showed a male predominance (77%) with younger age (<50 years) and minimal baseline cardiovascular risk. One population study (244 K individuals) documented high comorbidity: hypertension 57.6%, pre-existing CVD 39%, and obesity 31.1% (36).

## Discussion

This systematic review of 29 studies encompassing over 142.5 million individuals from 16 countries demonstrated that the available evidence does not support a significant causal association between nucleic acid-based COVID-19 vaccines and acute myocardial infarction in adults aged 18–80 years.

### Principal findings and population-level evidence

Most population-based studies have found no significant increase in AMI following vaccination. Two extensive British cohorts (45.7 and 46.2 million individuals) documented 37,915 and 13,609 AMI cases, respectively, without temporal or causal associations (20, 33). These findings were corroborated by US and French studies. Conversely, a Swedish cohort (8.07 million individuals) demonstrated protective effects (HR 0.81, 0.74–0.89), particularly after the third dose. This protective effect primarily reflects SARS-CoV-2 infection prevention rather than direct vaccine cardioprotection, as COVID-19 confers a 4–8-fold elevated AMI risk. While “healthy invitee bias” could theoretically contribute, the population-level design, comparable baseline risk profiles, and strengthened protection with successive doses argue against this mechanism of action. Vaccination cardioprotection primarily reflects the prevention of COVID-19 and its cardiovascular complications (21). The Malaysian study did not provide evidence of an increased myocardial infarction risk for nucleic acid vaccines (BNT162b2, ChAdOx1) and therefore does not constitute discordant evidence against the population-based registry findings for nucleic acid platforms (20, 31).

### Reconciling discordant population-level findings

Quality assessment revealed systematic differences favoring the Swedish findings. The Swedish study achieved comprehensive population capture through national health registries (>99% coverage), standardized outcome definitions, extensive confounding adjustment, and a 24-month follow-up (NOS 8/9, low bias). The Malaysian study relied on 50%–70% insurance coverage with less standardized coding and limited confounding adjustment (NOS 6.5/9; moderate bias) (21, 34).

The Swedish follow-up (December 2020–December 2022) included Omicron-dominant late periods with lower viral pathogenicity, whereas the Malaysian study (February–September 2021) encompassed Delta-dominant inflammation. Population differences included older Swedish cohort (median age ∼55 years, 15%–20% baseline CVD) vs. a younger Malaysian cohort (∼45–50 years, 8%–12% baseline CVD).

Sweden's universal system provides standardized diagnostics, eliminating detection bias, whereas Malaysia's mixed public/private system creates differential healthcare-seeking behavior, potentially inflating AMI ascertainment in vaccinated populations with better insurance coverage.

The Swedish approach used standardized troponin criteria, while the Malaysian approach used administrative coding, potentially misclassifying myocarditis as MI. Vaccine-associated myocarditis (1–10 per million) could inflate the number of “AMI” cases in administrative databases. Unmeasured confounding (smoking 20%–25%, occupational stress) could bias the Malaysian estimate upward by 20%, yielding a true IRR of ∼0.97 (protective effect). Genetic predisposition to thrombosis (e.g., MTHFR/PAI-1 polymorphisms) was identified in five fatal post-vaccination MI cases (48), although this does not establish causation, and similar genotypes are prevalent in background MI populations.

The methodological superiority of the Swedish study (NOS 8/9 vs. 6.5/9) justifies the 2× weighting. Consistency across multiple independent nations (Sweden, England, US, and France) strengthens the confidence that Malaysian findings reflect methodological artifacts rather than true causal effects. While the increased risk estimate in Malaysia cannot be dismissed, the available evidence suggests unmeasured confounding or outcome misclassification rather than a true vaccine effect.

### Case reports and quality context

Twelve case series documented 54 AMI cases (24–10 days post-vaccination), predominantly in young males with normal coronary arteries. The critical limitations included the absence of controls, reporting bias, and inability to distinguish temporal coincidence from causality. With >5 billion doses administered and a baseline AMI incidence of 200–500 per 100,000 person-years, thousands of cases necessarily occur through temporal coincidence. Quality assessment revealed that 51% of the case reports inadequately excluded alternative diagnoses. Temporal clustering in May–August 2021, coinciding with peak media attention, indicates publication bias rather than a true incidence increase. The proposed mechanisms (Kounis syndrome in two cases, VITT in one case, and coronary thrombosis in nine cases) are biologically plausible but lack population-level support. Individual case reports from diverse geographic regions, including the USA (42), Saudi Arabia (46), and others, documented similar temporal associations but shared the same fundamental limitations.

### Mechanistic evaluation

Four proposed mechanisms, Kounis syndrome, vaccine-induced thrombotic thrombocytopenia (VITT), inflammatory-induced thrombosis, and surveillance artifacts, require evaluation against population-level evidence. Kounis syndrome, while biologically plausible (predicted incidence 1–5 per million), demonstrates no clustering in atopic individuals (47). VITT confirms increased VTE (IRR 2.22) and thrombocytopenia (IRR 2.67) at population level (34); however, VITT-associated thromboses preferentially involve unusual sites (cerebral sinuses, portal vein), not epicardial coronaries; predicted coronary AMI incidence <1 per million with isolated case reports only (23, 24, 54). Inflammatory-induced thrombosis contradicts three key predictions: no temporal clustering (3,722 AMI within 28 days vs. 4,023 beyond), protective third-dose effect opposite to inflammation-driven hypothesis, and absent dose-response relationship; predicted incidence 5–50 per million with observed none to protective (34). Surveillance artifacts manifest as concentrated mid-2021 reports during the peak media attention. The VAERS PRR of 2.35 reflects reporting propensity, not causal risk, with only 41% of case reports adequately excluding alternative diagnoses (53). Although individually plausible, no mechanism operates at a sufficient frequency to generate detectable population-level increases. The most parsimonious explanation comprises rare genuine cases (<10 per million), thousands of expected temporal coincidences, substantial reporting bias, and population-based surveillance capturing no excess, an interpretation strengthened by consistency across diverse populations.

### Vaccine platform safety and dose-response

The mRNA vaccines demonstrated similar safety profiles in these studies. While ChAdOx1 exhibited specific thrombotic associations (thrombocytopenia IRR 2.67) (34), this did not translate to an elevated risk of AMI. Dose-response analysis provided reassuring evidence: Swedish data demonstrated maintained or enhanced protective effects through the third dose (HR: 0.81; 95% CI: 0.74–0.89), contradicting the cumulative adverse effect hypothesis (21). Complementing this, Yamin et al. found no elevated myocardial infarction risk following booster doses, with risk differences of 1.2 per 100,000 for the first monovalent booster, 2.3 for the second monovalent booster, and −4.9 for the bivalent booster, confirming safety even in vulnerable populations (38). French and Israeli booster studies similarly showed no increased risk (22, 49).

### Publication and reporting bias

Publication bias systematically enriches the literature on vaccine-associated AMI reports while suppressing cases excluding vaccine causation. Temporal clustering in mid-2021, rather than a uniform 2021–2025 distribution, suggests that publication practices fluctuate with attention cycles rather than true incidence changes.

Passive surveillance systems (VAERS) produce differential reports based on event-vaccine temporal associations. Clinicians were substantially more motivated to report AMI one-week post-vaccination than three months post-vaccination. Studies comparing passive vs. active surveillance consistently show that passive systems over-report temporally associated events by 2–5 folds. Population-based studies with active surveillance (Swedish, English, and French) using electronic health records captured all AMI cases independent of the vaccination status, consistently showing null or protective effects. This contrast reflects the magnitude of reporting bias rather than true causal differences.

### Risk-benefit analysis and clinical implications

COVID-19 mortality in unvaccinated individuals ranges from 1,000 to 3,000 per 100,000, with vaccines achieving 70%–95% hospitalization reduction and >90% mortality reduction. Even conservatively accepting the 16% relative risk increase in Malaysia, COVID-19 prevention yields substantial net benefits.

Evidence supports the continuation of COVID-19 vaccination without additional cardiovascular restrictions. Individuals with pre-existing cardiovascular disease are priority candidates because of the elevated risk of severe COVID-19. Post-vaccination surveillance (first 72 h) coupled with patient education is prudent and does not generate unnecessary vaccine hesitancy. Pharmacovigilance systems should maintain robust signal detection, as demonstrated by the timely identification of VITT.

### Future research

Prospective studies employing standardized Brighton Collaboration definitions, demographic stratification, and long-term follow-up beyond one year are warranted. Real-world effectiveness studies that simultaneously evaluate vaccination benefits and adverse events can refine population-specific risk-benefit estimates.

### Limitations of the systematic review

These findings must be interpreted within the context of important methodological limitations. This systematic review searched PubMed/MEDLINE, Cochrane CENTRAL, and Google Scholar without language restrictions and identified 1,328 records. Although these databases provide comprehensive coverage, specialized European pharmacovigilance repositories and gray literature were not systematically searched. A critical limitation is the inclusion of 12 case reports (41% of studies), which lack denominators, control groups, and standardized definitions, making them unsuitable for causal inference. Quality assessment revealed that 51% of inadequately excluded alternative diagnoses had temporal clustering in 2021, indicating publication bias. Although case reports merit signal detection, they provide no valid causal evidence. Population-based cohort studies employing active surveillance provide substantially stronger inferences and receive greater weight. The consistency across the eight independent registries spanning diverse healthcare systems substantially mitigated geographic publication bias concerns.

## Conclusions

Evidence from large-scale population-based studies encompassing 142.5 million individuals does not support a causal association between nucleic acid-based COVID-19 vaccines and acute myocardial infarction. Most studies demonstrated no association or protective effects, with a Swedish cohort showing protection (HR: 0.81; 95% CI: 0.74–0.89) after the third dose. Large-scale studies from England, the United States, France, Malaysia, Denmark, and Israel have consistently found no significant increase in AMI risk following mRNA or DNA-based vaccination.

Case reports require continued surveillance but carry critical limitations: absent controls, reporting bias, and inadequate alternative diagnosis exclusion, precluding causal inference. With over 5 billion doses administered and a baseline AMI incidence of 200–500 per 100,000 person-years, temporal coincidence accounts for thousands of cases.

The risk-benefit balance overwhelmingly favors vaccination. COVID-19 confers a 4–8-fold elevated AMI risk, far exceeding the theoretical vaccine risks. These findings support the continuation of existing immunization policies without additional cardiovascular restrictions.
