# Supplementary material for: Association between nucleic acid COVID-19 vaccines and acute myocardial infarction in adults: a systematic review
Source: Front Cardiovasc Med. 2026 Feb 12;13:1752169. doi: 10.3389/fcvm.2026.1752169 (PMC12936004; doi:10.3389/fcvm.2026.1752169)
Supplement: Supplementary file 1 [file Supplementaryfile1.pdf]

Supplementary Material 1: Detailed Search Strategy

Review Title: Association Between Nucleic Acid COVID-19 Vaccines and Acute Myocardial Infarction in Adults: A Systematic Review

Search Period: December 2020 to September 2025

Databases Searched: PubMed/Medline, Cochrane Library and Google Scholar

1. PubMed/Medline Search Strategy

Search Date: September, 2025

Step	Search Query	Results
**Concept 1: COVID-19 Vaccines**		
S1	"COVID-19 Vaccines"[Mesh]	8 288
S2	"mRNA Vaccines"[Mesh]	2 123
S3	"Vaccines, DNA"[Mesh]	587
S4	S1 OR S2 OR S3	8 372
S5	("COVID-19 vaccine*"[Title/Abstract] OR "SARS-CoV-2 vaccine*"[Title/Abstract] OR "mRNA vaccine*"[Title/Abstract] OR "RNA vaccine*"[Title/Abstract] OR "DNA vaccine*"[Title/Abstract] OR "BNT162b2"[Title/Abstract] OR "mRNA-1273"[Title/Abstract] OR "ChAdOx1"[Title/Abstract] OR "Pfizer"[Title/Abstract] OR "Moderna"[Title/Abstract] OR "AstraZeneca"[Title/Abstract])	6 943
S6	S4 OR S5	**8 924**
**Concept 2: Acute Myocardial Infarction & Cardiovascular Events**		
S7	"Myocardial Infarction"[Mesh]	5 050
S8	"Cardiovascular Diseases"[Mesh]	87 919
S9	"Coronary Thrombosis"[Mesh]	89
S10	S7 OR S8 OR S9	87 919
S11	("myocardial infarction"[Title/Abstract] OR "acute myocardial infarction"[Title/Abstract] OR "AMI"[Title/Abstract] OR "heart attack"[Title/Abstract] OR "cardiac arrest"[Title/Abstract] OR "coronary thrombosis"[Title/Abstract] OR "STEMI"[Title/Abstract] OR "NSTEMI"[Title/Abstract] OR "troponin elevation"[Title/Abstract])	10 862
S12	S10 OR S11	89 204
**Concept 3: Population/Age**		
S13	"Adult"[Mesh] OR "Middle Aged"[Mesh] OR "Aged"[Mesh] OR "Young Adult"[Mesh]	647 778
S14	("adult*"[Title/Abstract] OR "18-80 years"[Title/Abstract] OR "middle aged"[Title/Abstract] OR "elderly"[Title/Abstract])	202 398
S15	S13 OR S14	647 778
	**Final Combination**	
S17	(S6) AND (S12) AND (S13)	478
**Filters Applied**	Publication date: December 1, 2020 to September, 2025	

PubMed Search String:

(("COVID-19 Vaccines"[Mesh] OR "mRNA Vaccines"[Mesh] OR "Vaccines, DNA"[Mesh]) OR ("COVID-19 vaccine"[Title/Abstract] OR "SARS-CoV-2 vaccine*"[Title/Abstract] OR "mRNA vaccine*"[Title/Abstract] OR "RNA vaccine*"[Title/Abstract] OR "DNA vaccine*"[Title/Abstract] OR "BNT162b2"[Title/Abstract] OR "mRNA-1273"[Title/Abstract] OR "ChAdOx1"[Title/Abstract] OR "Pfizer"[Title/Abstract] OR "Moderna"[Title/Abstract] OR "AstraZeneca"[Title/Abstract])) AND (("Myocardial Infarction"[Mesh] OR "Cardiovascular Diseases"[Mesh] OR "Coronary Thrombosis"[Mesh]) OR ("myocardial infarction"[Title/Abstract] OR "acute myocardial infarction"[Title/Abstract] OR "AMI"[Title/Abstract] OR "heart attack"[Title/Abstract] OR "STEMI"[Title/Abstract] OR "NSTEMI"[Title/Abstract] OR "troponin"[Title/Abstract])) Filters: Publication date from December 1, 2020 to September 30, 2025)*

2. Cochrane CENTRAL Search Strategy

Step	Search Query	Results
#1	MeSH descriptor: [COVID-19 Vaccines] explode all trees	913
#2	("mRNA vaccine" or "DNA vaccine" or "Pfizer" or "Moderna" or "AstraZeneca"):ti,ab,kw	6 706
#3	MeSH descriptor: [Myocardial Infarction] explode all trees	15 815
#4	("myocardial infarction" or "heart attack" or "AMI"):ti,ab,kw	8 720
#5	#1 OR #2	5 156
#6	#3 OR #4	22 546
#7	#5 AND #6	102
Filters Applied	Limit to publication date 2020 to 2025	

3. Google Scholar Search Strategy

Search Iteration	Google Scholar Query	Estimated Results
**Search 1**	allintitle: infarction "COVID 19 vaccine"	19
**Search 2**	allintitle: infarction "SARS CoV 2"	72
**Search 3**	allintitle: myocardial infarction" "mRNA vaccine"	8
**Search 4**	myocardial infarction "DNA vaccine"	566
**Search 5**	allintitle: infarction AstraZeneca OR BNT162b2 OR "mRNA 1273" OR ChAdOx1 OR Pfizer OR Moderna OR AstraZeneca OR "covid 19 vaccine" OR "mRNA vaccine" OR "DNA vaccine"	36
**Search 7**	AMI SARS OR CoV OR 2 OR vaccination	~23
**Search 8**	allintitle: BNT162b2 cardiovascular OR myocardial	19
**Search 10**	allintitle: mRNA-1273 cardiovascular OR myocardial	5

PubMed Search String:

("COVID-19 vaccine" OR "SARS-CoV-2 vaccine" OR "mRNA vaccine" OR "RNA vaccine" OR "DNA vaccine" OR BNT162b2 OR "mRNA-1273" OR ChAdOx1 OR "Ad26.COV2.S" OR Pfizer OR Moderna OR AstraZeneca) AND ("myocardial infarction" OR "acute myocardial infarction" OR AMI OR "heart attack" OR STEMI OR NSTEMI OR "coronary thrombosis" OR troponin) AND (adult OR adults OR "young adult" OR "middle aged" OR elderly OR aged OR "18-80" OR "18 to 80")

Temporal Scope Justification:

Search period begins December 2020, coinciding with the FDA Emergency Use Authorization for the first COVID-19 vaccine (BNT162b2) and the initiation of mass vaccination campaigns globally.

End date September 2025 reflects the search completion date and captures the most recent evidence.

Search Terms Development:

MeSH terms were selected using the PubMed MeSH Browser to identify official controlled vocabulary for vaccines and cardiovascular outcomes.

Free-text terms captured vaccine product names (BNT162b2, mRNA-1273, ChAdOx1), trade names (Pfizer, Moderna, AstraZeneca), and specific outcome phenotypes (STEMI, NSTEMI, AMI).

Screening Process:

1. All retrieved citations from both databases were imported into a standardized screening software (RYYAN)
2. Duplicates were identified and removed using automated deduplication followed by manual verification
3. Title and abstract screening were performed independently by two reviewers (see Methods: Study Screening, Data Extraction, Quality Assessment)
4. Full-text articles meeting preliminary criteria underwent independent eligibility assessment by two reviewers

4. Strengths and Limitations of Search Strategy

Strengths:

- Two independent databases (PubMed + Google Scholar) minimize retrieval bias
- Explicit use of MeSH terms ensures reproducibility
- Gray literature inclusion reduces positive-result publication bias
- Comprehensive temporal coverage (5-year period)
- No language restrictions beyond English to capture international evidence

Limitations:

- PubMed indexing delays may result in recently published articles not being captured

- Google Scholar's algorithm is proprietary and non-transparent, potentially limiting reproducibility of Google Scholar results
- Truncation and Boolean operators function differently between platforms, requiring platform-specific query construction
