# Supplementary material for: Association between nucleic acid COVID-19 vaccines and acute myocardial infarction in adults: a systematic review
Source: Front Cardiovasc Med. 2026 Feb 12;13:1752169. doi: 10.3389/fcvm.2026.1752169 (PMC12936004; doi:10.3389/fcvm.2026.1752169)
Supplement: Supplementary file 2 [file Supplementaryfile2.docx]

**Supplementary Material 2: PRISMA 2020 Checklist for Systematic Reviews**

Review Title: Association Between Nucleic Acid COVID-19 Vaccines and Acute Myocardial Infarction in Adults: A Systematic Review

Authors: Dalia I. Castellanos-Hernández, Miguel A. Mayoral-Chávez, Carlos A. Matías-Cervantes, and Juan Alpuche.

PROSPERO Registration ID: CRD420251112911 (ID: 1112911)

Date Completed: December 29, 2025

PRISMA 2020 Checklist

| **#** | **Checklist Item** | **Location in Manuscript** |
| --- | --- | --- |
| **TITLE** |  |  |
| 1 | Identify the report as a systematic review | Title page; Abstract |
| **ABSTRACT** |  |  |
| 2 | See PRISMA 2020 for Abstracts checklist | Abstract section (includes PROSPERO ID: 1112911) |
| **INTRODUCTION** |  |  |
| 3 | Describe the rationale for the review in the context of existing knowledge | Introduction, paragraphs 3-5 |
| 4 | Provide an explicit statement of the objective(s) or question(s) the review addresses | Introduction, final paragraph |
| **METHODS** |  |  |
| 5 | Specify the inclusion and exclusion criteria for the review; ideally using the PICOS framework | Methods Section 2.2: Study Selection Criteria (PICOS Framework) |
| 6 | Specify all databases and registers searched, search date ranges, and any restrictions | Methods Section 2.1: Information Sources; Supplementary Material 1 |
| 7 | Identify all search strategies used, including search terms, Boolean operators, etc. | Supplementary Material 1: Detailed Search Strategy |
| 8 | Specify the method used to decide which studies to include in the review | Methods Section 2.3: Study Screening |
| 9 | Specify the method used to extract data from reports | Methods Section 2.3: Data Extraction subsection |
| 10 | Specify how you assessed the risk of bias in the included studies | Methods Section 2.3: Quality Assessment subsection |
| 10a | Specify how you assessed the risk of bias arising from missing results | Methods Section 2.3; Discussion regarding pharmacovigilance |
| 11a | Specify the effect measures used in each study | Results section; Tables 1-3 |
| 11b | Specify whether you calculated a pooled effect estimate and its 95% CI | Methods Section 2.4: Data Synthesis (qualitative synthesis only) |
| 11c | Describe methods used to evaluate certainty of evidence | Methods Section 2.5: Evidence Certainty |
| 12 | Describe the methods used to evaluate heterogeneity between studies | Methods Section 2.4: Heterogeneity Exploration |
| 13 | Describe analyses conducted to explore possible causes of heterogeneity | Methods Section 2.4: Heterogeneity Exploration (stratified analyses) |
| 14 | Describe methods used to assess reporting bias | Methods Section 2.5: Publication Bias Assessment |
| 15 | Describe how you addressed reviews published in duplicate formats | Methods Section 2.3: Study Screening (duplicate removal) |
| **RESULTS** |  |  |
| 16 | Provide numbers of studies screened, assessed, and included | Results Section 3.1; Figure 1 (PRISMA Flow Diagram) |
| 17a | Present summary data and effect estimates for each study | Results Sections 3.2-3.4; Tables 1-7 |
| 17b | Present results of risk of bias assessment | Results Section 3.3; Table with NOS/quality scores |
| 17c | Present results of all statistical analyses performed | Results Sections 3.4-3.7; detailed tables |
| 18 | Report results of assessments of reporting bias | Results Section 3.7: Publication Bias; Discussion |
| 19 | Present results of analyses examining heterogeneity | Results Section 3.5: Stratified Analyses |
| 20 | Present results of analyses investigating causes of heterogeneity | Results Section 3.5; Discussion |
| 21 | Present findings of certainty of evidence assessment | Discussion section; GRADE assessment integrated |
| **DISCUSSION** |  |  |
| 22 | Provide a general interpretation of the findings | Discussion Sections 1-3 |
| 23 | Discuss implications for practice, policy, and future research | Discussion Sections 6-7 |
| 24 | Discuss limitations of the review | Discussion Section 5: Limitations |
| **OTHER INFORMATION** |  |  |
| 25 | Provide registration information for the review | Methods Section 2.0: PROSPERO ID: 1112911 |
| 26 | Indicate where the review protocol can be accessed | Methods Section 2.0 (Available via PROSPERO) |
| 27 | Describe sources of financial or non-financial support | Acknowledgments/Funding section |
